# Lord Rosebery's Work

**Published:** 1889-08-03

**Authors:** 


### The Hospital

A Weekly Institutional Journal of

Science, Medicine, Nursing, and Philanthropy.

*For Hospitals, Asylums, Medical Practitioners, Students, Nurses, Families, Parishes, Congregations, and the Charitable Public.*

Vol. VI. No. 149.] [August 3, 1889.

### Lord Rosebery's Work.

Lord Rosebery shows a highly satisfactory appreciation of many of the difficult questions that arise as the result of the massing together of such enormous numbers of human beings as we see in London. The disposal of sewage is one of those questions; and it must be admitted that there is nothing about sewage and its disposal to excite the admiration of the orator or to lend wings to the flights of the poet. But for all that, the sewage of London is a life and death question: much more immediately urgent than the conquering of the Mahdi and his dervishes, and quite as serious as a threatened invasion of Canada by the United States. Bacterial pathology has shown in the plainest and most unmistakeable ways that sewage contains a variety of living creatures, each of which is capable of astonishing rapidity of reproduction; and the reproduction of which in the human system is the prompt cause of illnesses of the most dangerous character, which very frequently end in death. If ten thousand mad dogs were kennelled in London, and kennelled so carelessly that a few scores or hundreds of them might break out at any moment and devastate the town, we should all be in a condition of hourly and terrified alarm. The sewage which lies beneath our houses, floods our street drains, and is delivered by mains into the open ocean, is quite as dangerous and much more subtle than ten thousand mad dogs would be if kennelled in our midst.

Last Friday, at the Wimbledon Sewage Farm, Lord Rosebery and many other well-known persons witnessed a demonstration of the "Amines process" for the treatment and purification of sewage. The public is a little weary of these almost infinitely multiplied "processes" of "treatment and purification;" and the general impatience is not at all unnatural under the circumstances. But let every intelligent reader remember that the sewage problem is still unsolved; and that on its solution depend the lives and health, not merely of Londoners and town-dwellers in general, but of himself, his children, his friends, and all that he holds dearest and most valuable in the world. That the public should be indifferent is a great calamity. Life is very simple in the remote farmhouse or the country village; but wherever people are massed together in numbers, there all kinds of difficult problems must inevitably arise; and if the people are capable they will face every difficulty with intelligent courage, and deal with it effectively. If, on the other hand, they are too indolent or too stupid to take the steps demanded by the occasion, difficulties will accumulate, and the penalties must be paid in pestilence, fever and death.

The sewage problem is still unsolved, we have said, and, therefore, it is the clear duty of the London County Council, as of other municipal bodies, to be on the alert, if by any means they may discover a solution. The "Amines process" has much to say for itself theoretically, and Lord Rosebery did well to go to Wimbledon and see for himself what it can accomplish practically. It may be taken for granted that the president of the London County Council is not ignorant of practical methods of dealing with town sewage. For many years he has taken a very active part in all the important public business of the city of Edinburgh, which may be called his native city. That city solved the sewage problem for itself long ago, in what is perhaps the only really final and effectual way. The Craigentinny meadows receive all, or nearly all, of the Edinburgh sewage, which is distributed to their various parts periodically as they are fit to receive it. About those meadows and the sewage nothing need be said except this, that all the latter is utilised to the great benefit of the former, whilst the cost of removal is quite inconsiderable. Edinburgh, in fact, deals with its sewage in the only natural and rational way, by returning it to the land. With the Edinburgh method Lord Rosebery is, no doubt, perfectly familiar.

What can London do with its sewage which shall make it as harmless and as useful as the sewage of Edinburgh is said to be? It can return it to the land like Edinburgh. But that is a method of dealing with it which has never commended itself to the metropolitan official mind. "Treatment" of some kind, "purification" by some means, are the objects that our chemists and engineers aim at. In the absence of the best and most rational method—viz., return to the soil—let us have "treatment" and "purification" by all means. But it must be effective treatment and real purification. What we all have a right to complain of is that in spite of the thousand and one new methods that have been tried and the public money that has been spent, the river, which may be called our sewage barometer, still marks not "fair," nor even "change," but "rain," "much rain," and "storm;" in other words, judging by the odour of the river, we are as far off from a perfect method of sewage purification as if no single experiment had ever been made.

Will the "Amines process" bring us any nearer the goal of effective sewage purification? In order to answer that question another may be asked: What is the chief point to be aimed at in the purification of sewage? Thanks to the researches of modern physiological scientists, we are able to answer the question with absolute precision. We know exactly what we want to do. The element in the sewage which makes it dangerous to life and health is that familiar disease germ, the bacterium. There are many varieties of these minute organisms, some of which give rise to typhoid fever, some to diphtheria, some to small-pox, and so on. If these little creatures can be killed effectually, those diseases will to a large extent disappear from amongst us. But can they be killed for certain by any known process; above all, can the innumerable hordes of them which live and thrive and increase by myriads daily in the London sewage be certainly and universally destroyed? The believers in the "Amines process" say they can; and Dr. Klein, F.R.S., one of our most trusted scientists in the field of bacteriology, confirms their statement.

The experiments made by Dr. Klein, on which he bases his reports, are easily understood. He first took a bottle of sewage in its native state, bottled and sealed for the purpose under the superintendence of the Town Council of West Ham. To a portion of perfectly sterile nutrient gelatine on a cultivation plate he added a small quantity of the untreated sewage. He reports that such was the potency of the sewage that on the fourth day the number of colonies of all kinds of bacteria on the cultivation plate was "far too great to be counted." In other words, the poison contained in such sewage was of the most deadly character. Dr. Klein, for purposes of comparison, was very desirous of counting the number of bacteria in each cultivation, and therefore, instead of using the sewage in its natural condition, he diluted it with two thousand times its own bulk of pure distilled water. This, when added to the sterile gelatine on the cultivation plate, was further diluted until the degree of dilution was as 100,000 of water and gelatine to one of pure sewage. At the end of six days 24 bacterial colonies could be counted on the cultivation plate, containing in the aggregate 2,400,000 bacteria. Three more bottles of sewage were experimented on in a similar way, each of them having been taken at a different stage of the purifying process. The second bottle, which is called "Press Liquor," was so much purer than the original sewage, that instead of showing nearly two and a half millions of dangerous bacteria there were only 650 altogether on the plate. The third bottle, called "sludge," was still further purified, and the plate revealed only 400 bacteria. The fourth bottle, called "subsidence effluent," and which consisted of the purified sewage ready to be discharged into river or sea, showed no bacteria at all.

If Dr. Klein's experiments really indicate the actual degree of purification which can be effected in the whole bulk of the London sewage, the "Amine's process" will be entitled to rank first among all the measures which have hitherto been devised for the treatment of sewage in large quantities. That, however, remains to be seen. The experiments at Wimbledon were held by experts to be highly satisfactory. Whilst no method of dealing with sewage, except that which returns it to the soil, can be considered final, yet, if a means for cleansing and keeping the Thames and other large streams fairly pure has at last been discovered, all the world will be grateful. We await with much interest the further developments of the new process.

